# Native strains of *Beauveria bassiana* for the control of *Rhipicephalus sanguineus* sensu lato

**DOI:** 10.1186/s13071-015-0693-9

**Published:** 2015-02-05

**Authors:** Claudia Cafarchia, Davide Immediato, Roberta Iatta, Rafael Antonio Nascimento Ramos, Riccardo Paolo Lia, Daniele Porretta, Luciana Aguiar Figueredo, Filipe Dantas-Torres, Domenico Otranto

**Affiliations:** Department of Veterinary Medicine, University of Bari, Bari, Italy; Department of Environmental Biology, University “La Sapienza”, Rome, Italy; Department of Immunology, Centro de Pesquisas Aggeu Magalhães (Fiocruz-PE), Recife, Pernambuco Brazil

**Keywords:** *Beauveria bassiana*, Biological control, *Rhipicephalus sanguineus* s.l., Entomopathogenic fungus, Vector borne-diseases

## Abstract

**Background:**

*Rhipicephalus sanguineus* sensu lato ticks are widespread worldwide due to their adaptability to survive under different environmental conditions. They may act as vectors of a wide range of pathogens to humans and animals and their control is based on the use of chemical products on dogs and in the environment. Alternative control strategies, such as the use of entomopathogenic fungi as bio-control agents have also been investigated. The ability of native strains of *Beauveria bassiana* sensu lato in causing mortality in different tick species (e.g., *Amblyomma cajennense* and *Rhipicephalus microplus*) has been demonstrated. However, limited studies have assessed the use of *B. bassiana* for the control of *R. sanguineus* s.l. and none of them have employed native strains of this fungus. Here we investigated the pathogenicity of a native strain of *B. bassiana* (CD1123) against all developmental stages of *R. sanguineus* s.l..

**Methods:**

Batches of eggs, larvae, nymphs and adult ticks were immersed in a suspension of 10^7^ conidia/ml of *B. bassiana* s.l., isolated from a *R. sanguineus* s.l. engorged female. All treatment and control groups were observed for 20 days, and the biological parameters (i.e., mortality, hatching, moulting percentage, pre-oviposition period, oviposition period and rate, eggs production efficiency, reproductive efficiency and fitness indexes) were assessed.

**Results:**

The effect of the *B. bassiana* strain tested herein on eggs, larvae, nymphs and adults showed a significantly higher mortality than those of the control groups (p < 0.05) at 5 days post-infection. No infected eggs hatched and no infected larvae moulted. Only 15% of infected nymphs moulted into adults. All biological parameters of treated groups differed significantly (p < 0.001) from those of control groups.

**Conclusions:**

This study demonstrates that a suspension containing 10^7^ conidia/ml of a native *B. bassiana* strain is highly virulent towards all life-cycle developmental stages of *R. sanguineus* s.l. and may be of potential interest as a biological control agent against these ticks.

## Background

Ticks are major vectors for a wide range of pathogens (e.g., viruses, bacteria and protozoa) of medical and veterinary concern. Among ixodid ticks, *Rhipicephalus sanguineus* sensu lato (s.l.) is vector of agents causing the so-called tick-borne diseases (TBDs) in dogs (e.g., *Ehrlichia canis*, *Babesia vogeli* and *Hepatozoon canis*) and humans (e.g., *Rickettsia conorii* and *Rickettsia rickettsii*) [1]. Due to their ability to survive under different climatic conditions and ecological niches, these ticks have a cosmopolitan distribution and their control is still a major challenge for veterinarians and pet owners. The main line of defence against ticks is the use of chemicals [2], although this approach may present disadvantages such as the development of resistance, human and animal toxicity and environmental pollution [3,4]. For example, the resistance of *R. sanguineus* s.l. to some synthetic acaricides such as amitraz [5] has spurred the interest of the scientific community in developing alternative methods to their control [2].

Entomopathogenic fungi have been investigated for their potential in the biological control of these arthropods due to their ability to penetrate the integument of ticks [6]. In particular, *Beauveria bassiana* s.l. and *Metarhizium anisopliae* s.l. were effective in controlling several tick species including *Rhipicephalus microplus*, *R. sanguineus* s.l., *Dermacentor nitens* and *Amblyomma cajennense* s.l. [6]. The susceptibility to fungi might vary according to tick species and population as well as to fungal strain [6-9]. For example, in studies of pathogenicity recorded for *B. bassiana* s.l. and *M. anisopliae* s.l., the native strains (i.e., isolated from the environment or naturally infected ticks) have been shown to be more virulent [6-9]. However, of the few studies available in the literature on the use of *B. bassiana* s.l. as bio-control agent against *R. sanguineus* s.l. [10-13], none have tested native strains of this fungus. Therefore, the aim of this study was to investigate the *in vitro* efficacy of a native strain of *B. bassiana* on eggs, adults and immature stages (i.e., larvae, nymphs) of *R. sanguineus* s.l..

## Methods

### Tick samples

In April 2012, engorged female ticks were collected from clinically healthy dogs from a private dog shelter in Putignano (40° 50’ N, 17° 07’ E, 372 m a.s.l.), province of Bari, southern Italy. Ticks were identified based on morphological and genetic data as *Rhipicephalus* sp 1 (*R.* sp.1) [14]. These engorged females were employed in the bioassay and for tick rearing. Tick specimens were maintained under controlled conditions of temperature (27 ± 1°C), relative humidity (RH 80 ± 5%) and photoperiod (12 h light, 12 h dark) to produce eggs. Newly hatched larvae were allowed to feed on rabbits until detachment. Then, they were maintained under controlled conditions (as above) until moulting into nymphs. Afterwards, hatched nymphs were allowed to feed on rabbits until detachment and kept at the same conditions as previously described, until moulting into adults. Finally, adults were placed on rabbits for feeding and mating, and engorged females were individually transferred to glass tubes to lay eggs.

### *Beauveria bassiana* strain origin and identification procedures

Native *B. bassiana* isolates were obtained from naturally infected ticks collected in a private dog shelter in Putignano, province of Bari, southern Italy (see above). Naturally infected ticks were cultured onto Sabouraud dextrose agar with chloramphenicol (0.5%) (SDA-Liofilchem Diagnostici®, Roseto degli Abruzzi, Italy) and incubated at 25°C for 15 days. For each positive sample, the colonies were sub-cultured on SDA plates and identified based on their morphology by microscopic examination of the hyphae and conidia, as described elsewhere [15,16]. The identification was also confirmed through molecular analysis [7]. Briefly, four *Beauveria* isolates were cultured in SDA for 4 days at 25°C and extraction of genomic DNA was performed from the cultured isolates using the ArchivePure DNA Yeast kit (5-Prime Inc., USA). The internal transcribed spacer 1 (ITS1) and ITS2 regions and the 5.8S ribosomal DNA (rDNA) region of the fungi were amplified by using the primers ITS1 and ITS4 [7].

Polymerase chain reaction (PCR) was carried out in a 50 μL final volume including 2 μL template DNA (5–10 ng), 10 mM Tris–HCl (pH 8.3), 50 mM KCl, 2.5 mM MgCl_2_, 250 μM of each dNTP, 1 μM of each primer and 1.25 U of AmpliTaq Gold (Applied Biosystems). The reaction conditions were as follows: 94°C for 5 min for initial denaturation and 35 cycles of 30 s at 94°C, 30 s at 58°C and 30 s at 72°C, with a final extension at 72°C for 10 min. A blank, no template, control was included in the PCR. The products were analyzed in 1.5% ethidium bromide-stained agarose gel (Gellyphor, EuroClone, Milan, Italy). Purified PCR products were sequenced using the Taq DyeDoxy Terminator Cycle Sequencing Kit (v.2, Applied Biosystems) in an automated sequencer (ABI-PRISM 377). Sequences were aligned using ClustalW program and compared among them and with those available in GenBank database by Basic Local Alignment Search Tool (BLAST – http://blast.ncbi.nlm.nih.gov/Blast.cgi).

### *Beauveria bassiana* conidial infection suspension

The fungi were maintained on potato dextrose agar (PDA) and kept at 4°C. The conidial infection suspension (CIS) of *B. bassiana* was obtained by growing the fungi on 10 Petri dishes containing PDA for 3 weeks at 25°C. Conidia were harvested by washing the Petri dishes with sterile distilled water containing 0.1% Tween 80 and by transferring them to an assay tube. Turbidity was adjusted spectrophotometrically (Biosan DEN 1) to an optical density of 4.5 McFarland, corresponding to 1–5 × 10^7^ conidia/ml. The amount of conidia was evaluated by quantitative plate counts of colony forming unit (CFU)/ml in SDA.

### Laboratory bioassays

A total of 1120 tick specimens (i.e., 160 females, 640 larvae, and 320 nymphs) and about 4000 eggs were tested. All bioassays were composed of 2 groups [control group (CG) and test group (TG)] of ticks for each life stage (i.e., adult females, larvae, nymphs and eggs). Each group was composed by four subgroups of variable tick number, with a homogeneous weight (see below).

#### Adult females

Ticks, both engorged and unfed, were subjected to the adult immersion test (AIT) [17]. Each group was composed of four subgroups of five ticks each. Ticks in the TG were immersed in 1 ml of CIS for 3 min, whereas those of CG were immersed in sterile distilled water plus 0.1% Tween 80. After treatment, the specimens were placed in Petri dishes, labelled and kept in an incubator under controlled conditions (27 ± 1°C and RH 80 ± 5%) to lay eggs.

The female mortality was evaluated daily for 20 days. In addition, the following biological parameters were assessed for engorged females: pre-oviposition weight, pre-oviposition period (number of days from collection to the beginning of oviposition), oviposition period (number of days from the beginning to the end of oviposition), oviposition rate (proportion of engorged females that oviposited), weight of eggs, number of eggs, egg hatch rate (mean value of visual evaluation performed by three examiners), egg incubation period (number of days from the beginning of oviposition to the hatching of the first larva), egg production efficiency (weight of eggs/weight of the engorged female × 100), reproductive efficiency index (number of eggs/weight of the engorged female), and reproductive fitness index (number of eggs that hatch into larvae/weight of the engorged female).

#### Eggs

The eggs produced by CG females were collected and divided into batches of 10 mg (i.e., about 250 eggs). They were placed in individual glass tubes, closed with a cotton plug, and maintained in an incubator under controlled conditions (see above). The bioassay was composed of four test tubes for each CG and TG, containing an egg batch of 10 mg each tube. The eggs were immersed in 1 ml of the CIS or control solution following the same methodology applied to treat the engorged females (see above). After 3 min, the CIS was discarded and the test tube was sealed with a cotton plug. The tubes were maintained at 27 ± 1°C and RH 80 ± 5%. The incubation period and hatching rate were estimated daily by three examiners, under a stereomicroscope.

#### Larvae and nymphs

*R. sanguineus* s.l. engorged and unfed larvae and nymphs were treated following the same protocol used for adult females. Each group was composed of four subgroups of 20 larvae/10 nymphs each. Ticks in the TG were immersed in 1 ml of CIS for 3 min, whereas those of CG, in sterile distilled water plus 0.1% Tween 80. The mortality rate and moulting percentage (only for engorged specimens) were estimated daily for 20 days under a stereomicroscope by three examiners.

### Data analysis

Each bioassay was repeated twice, and the results were reported as mean values. All parametric (pre-oviposition weight, pre-oviposition period, oviposition period, weight of eggs, number of eggs, eggs incubation periods, egg production efficiency, reproductive efficiency and fitness indexes) and non-parametric (mortality, oviposition rate, egg hatching rates and moulting rate) data of CG and TG were compared. The parametric data were analysed using analysis of variance followed by the Student’s *t*-test, with 5% significance (p < 0.05). Conversely, the non-parametric data were analysed using chi-square test, with 5% significance (p < 0.05) [18].

## Results

The four *Beauveria* isolates were morphologically identified as *B. bassiana.* Sequencing of PCR amplicons (530 bp) from individual DNA samples of four *Beauveria* isolates revealed one ITS sequence type that matched with previously determined sequences (accession nos. KC753391, KC753388, KC753385, DQ364698, AY532013) with 100% of identity. One nucleotide sequence representative for all amplicons sequenced, has been deposited in the GenBank database under the accession number KP216528. The characterized strain (code CD1123) is stored at −80°C in the fungal collection of the mycology section at the Department of Veterinary Medicine, University of Bari, Italy.

**Figure 1:**
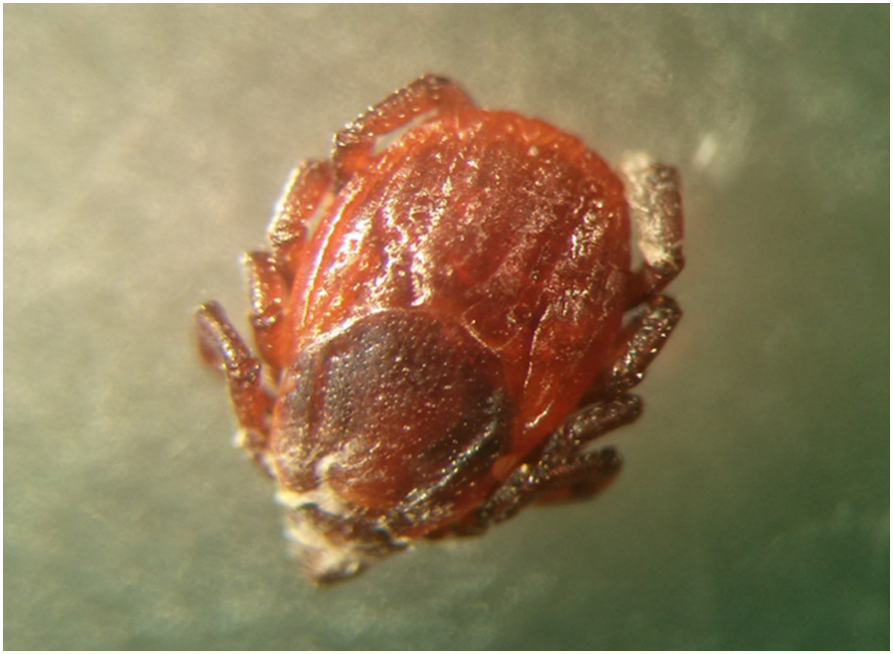
**Unfed female of** ***Rhipicephalus sanguineus*** **s.l. at 3 days post infection with** ***Beauveria bassiana.***

**Figure 2:**
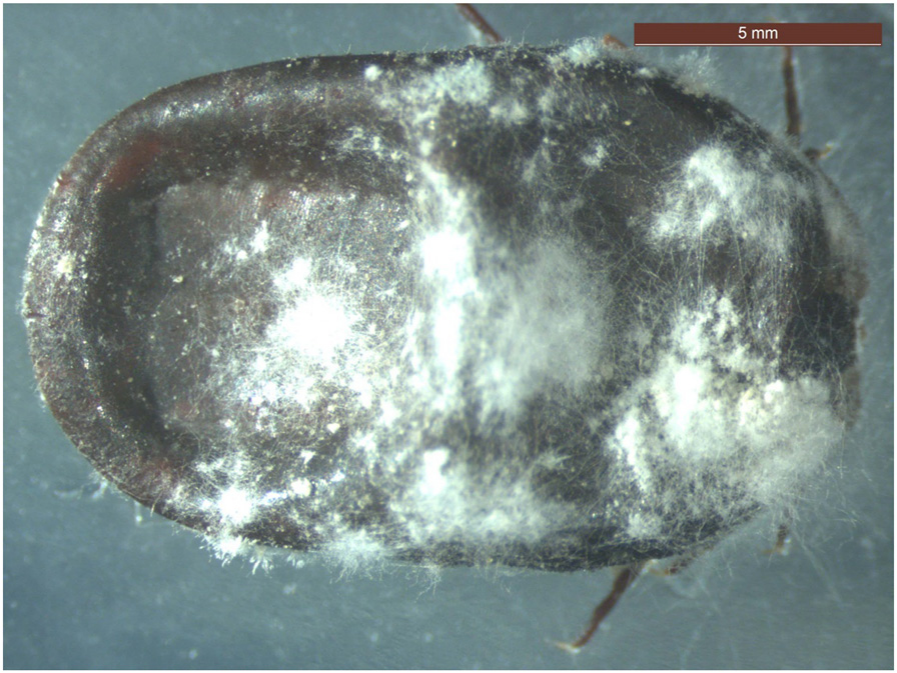
**Mycelium and conidiophores of** ***Beauveria bassiana*** **on an engorged female of** ***Rhipicephalus sanguineus*** **s.l. at 10 days post infection.**

**Figure 3:**
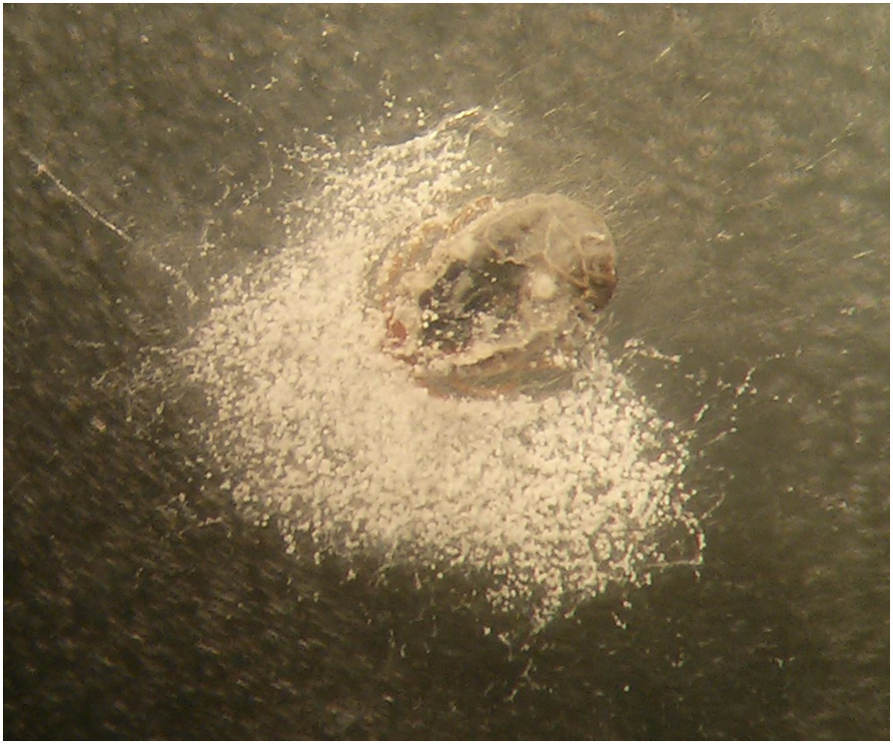
**Mycelium and conidiophores of** ***Beauveria bassiana*** **on an engorged larva of** ***Rhipicephalus sanguineus*** **s.l. at 20 days post infection.**

**Figure 4:**
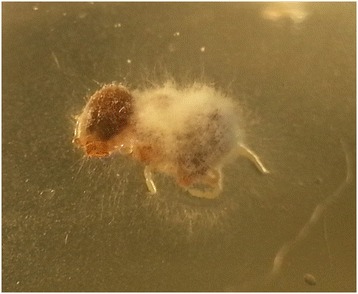
**Mycelium without conidiophores of** ***Beauveria bassiana*** **on an engorged nymph of** ***Rhipicephalus sanguineus*** **s.l. at 15 days post infection.**

The *in vitro* effect of *B. bassiana* on engorged and unfed females, eggs, larvae and nymphs is shown in Figures 1, 2, 3, 4 and Tables 1, 2, 3, 4, 5. White fungal mycelium started to emerge and sporulate on the surface of all the developmental stages of *R. sanguineus* s.l. from TG, 3 days post-infection (PI, Figure 1). Fertile conidiophores were present in all the developmental stages of infected ticks (Figures 2, 3) with the exclusion of nymphal stages (Figure 4). No significant difference in the initial weight of each stage was recorded in TG and CG before the exposure to *B. bassiana*. All biological parameters of TG differed significantly (p < 0.001) from those of CG (Tables 1, 2, 3, 4, 5). Briefly, a higher mortality was observed at day 5 PI in all the developmental stages of *R. sanguineus* s.l. from TG than those from CG (Tables 1, 2, 3, 4). The mortality rate reached 100% within 15 days PI in engorged females, engorged and unfed larvae. The highest mortality rate registered for engorged nymphs was 85% at 10 days PI (Table 4). The reproductive parameters (i.e., pre-oviposition and oviposition periods, oviposition rate, weight and number of eggs, egg incubation period, egg production efficiency, reproductive efficiency and fitness indexes) of engorged females from TG were significantly reduced (p < 0.001) (Table 5). No egg or larva from TG hatched or moulted, respectively (Tables 2 and 3), and only 15% of TG nymphs moulted into adults (Table 4).

**Table 1:** **Effects of a native strain of** ***Beauveria bassiana*** **on engorged and unfed females of** ***Rhipicephalus sanguineus*** **sensu lato**

**Mortality at**	**Engorged females Pos/Tot (%)**		**Unfed females Pos/Tot (%)**	
	**Treated**	**Control**	**Treated**	**Control**
Day 5	24/40 (60%)^a^	2/40 (5%)^a^	8/40 (20%)^m^	0/40 (0%)^m^
Day 10	38/40 (95%)^b^	2/40 (5%)^b^	16/40 (40%)^n^	2/40 (5%)^n^
Day 15	40/40 (100%)^c^	10/40 (25%)^c^	30/40 (75%)^o^	4/40 (10%)^o^
Day 20	40/40 (100%)^d^	25/40 (62%)^d^	34/40 (85%)^p^	6/40 (15%)^p^

^a-p^ = Chi square test, P < 0.001.

The statistically significant differences are indicated with the same superscript letters.

**Table 2:** **Effect of a native strain of** ***Beauveria bassiana*** **on eggs of** ***Rhipicephalus sanguineus*** **sensu lato**

**Parameters**	**Eggs**	
	**Treated**	**Control**
Incubation period (days)	0 ± 0^a^	21 ± 0.8^a^
Egg hatch rate, Pos/Tot (%)	0/2000 (0%)^b^	1836/2000 (91.8%)^b^

^a^ = Chi square test, P < 0.001; ^b^ = Student’s *t*-test, P < 0.001.

The statistically significant differences are indicated with the same superscript letters.

**Table 3:** **Effect of a native strain of** ***Beauveria bassiana*** **on engorged and unfed larvae of** ***Rhipicephalus sanguineus*** **sensu lato**

**Mortality at**	**Engorged larvae Pos/Tot (%)**		**Unfed larvae Pos/Tot (%)**	
	**Treated**	**Control**	**Treated**	**Control**
Day 5	76/160 (47.5%)^a^	0/160 (0%)^a^	88/160 (55%)^f^	16/160 (10%)^f^
Day 10	158/160 (98.7%)^b^	0/160 (0%)^b^	134/160 (83.7%)^g^	16/160 (10%)^g^
Day 15	158/160 (98.7%)^c^	8/160 (5%)^c^	160/160 (100%)^h^	22/160 (13.7%)^h^
Day 20	158/160 (98.7%)^d^	12/160 (7.5%)^d^	160/160 (100%)^i^	22/160 (13.7%)^i^
Moulting rate	2/160 (1.2%)^e^	148/160 (92.5%)^e^	-	-

^a-i^ = Chi square test, P < 0.001.

The statistically significant differences are indicated with the same superscript letters.

**Table 4:** **Effect of a native strain of** ***Beauveria bassiana*** **on engorged and unfed nymphs of** ***Rhipicephalus sanguineus*** **sensu lato**

**Mortality at**	**Engorged nymphs Pos/Tot (%)**		**Unfed nymphs Pos/Tot (%)**	
	**Treated**	**Control**	**Treated**	**Control**
Day 5	22/80 (27.5%)^a^	0/80 (0%)^a^	12/80 (15%)^f^	0/80 (0%)^f^
Day 10	68/80 (85%)^b^	0/80 (0%)^b^	26/80 (32.5%)^g^	4/80 (5%)^g^
Day 15	68/80 (85%)^c^	0/80 (0%)^c^	46/80 (57.5%)^h^	4/80 (5%)^h^
Day 20	68/80 (85%)^d^	0/80 (0%)^d^	70/80 (87.5%)^i^	6/80 (7.5%)^i^
Moulting rate	12/80 (15%)^e^	80/80 (100%)^e^	-	-

^a-i^ = Chi square test, P < 0.001.

The statistically significant differences are indicated with the same superscript letters.

**Table 5:** **Effects of a native strain of** ***Beauveria bassiana*** **on biological parameters of engorged females of** ***Rhipicephalus sanguineus*** **sensu lato**

**Biological parameters**	**Treated (n = 40)**	**Control (n = 40)**	**Statistics**
Female pre-oviposition weight (mg)	159.2 ± 71.9	164.1 ± 77.8	Student’s *t*-test, P = 0.77
Pre-oviposition period (days)	3 ± 2.8	4.9 ± 1.7	Student’s *t*-test, P < 0.001
Oviposition period (days)	2.6 ± 3.5	11.4 ± 2.1	Student’s *t*-test, P < 0.001
Oviposition rate, Pos/Tot (%)	18/40 (45%)	33/40 (82.5%)	Chi square test, P = 0.001
Weight of eggs for single female (mg)	4.9 ± 7.6	52.5 ± 18.2	Student’s *t*-test, P < 0.001
Number of eggs for single female	124.1 ± 189.8	1312 ± 453.3	Student’s *t*-test, P < 0.001
Egg hatch rate, Pos/Tot (%)	97.6/124.1 (78.7%)	1293/1313 (98.5%)	Chi square test, P < 0.001
Egg incubation period (days)	7.6 ± 14.2	27 ± 1.6	Student’s *t*-test, P < 0.001
Egg production efficiency	4.1 ± 6.8	37.3 ± 14.8	Student’s *t*-test, P < 0.001
Reproductive efficiency index	1.0 ± 1 7	9.3 ± 3.7	Student’s *t*-test, P < 0.001
Reproductive fitness index	0.7 ± 1.5	12.1 ± 1.2	Student’s *t*-test, P < 0.001

Otherwise indicated, numbers are reported as mean ± standard deviation.

## Discussion

The present study shows, for the first time, that a native strain of *B. bassiana* is highly virulent towards *R. sanguineus* s.l.. Indeed, CIS containing 10^7^ conidia/ml was highly pathogenic to all tick developmental stages affecting the vitality but also the egg laying, the larval hatching and larval and nymphal moulting. In particular, an increase in the mortality rate of all tick stages, a reduction of larval and nymphal moulting rate, egg production and hatching rate, were observed. The mortality rate herein reported for all developmental stages of *R. sanguineus* s.l. (i.e., 100% within 15 days)*,* except nymphs, is the highest ever recorded in the international literature [7-11,13,19-23]. Indeed, *B. bassiana* and *Metarhizium* spp. were used as control agents for different species of ticks (e.g., *R. microplus*, *R. annulatus*, *Hyalomma excavatum*, *R. sanguineus* s.l., *Rhipicephalus appendiculatus*, *Amblyomma variegatum*) displaying a mortality rate lower than 100% within 20 days, according to each developmental stage [7-11,13,19-23]. The results indicate a high pathogenicity of the native *B. bassiana* strain used herein against *R. sanguineus* s.l., therefore representing a potential candidate agent for controlling these ticks.

The reduced mortality of nymphs in comparison with other stages might be due to different composition of host cuticle, as previously reported for unfed nymphs of *R. sanguineus* s.l. treated with *M. anisopliae* [6,21]. Indeed, the conidial germination and the formation of appressoria are important events in the interactions between entomopathogenic fungi and their arthropod hosts [20-24]. The lipid composition of tick epicuticles selectively affects the germination of conidia of entomopathogenic fungi and thus the mortality of ticks [20-24]. Adhesion, germination and production of conidia are recognized as the main virulence factors favouring the infection process of entomopathogenic fungi against arthropods [20,24]. In particular, the presence of conidiophores producing conidia on the hosts surface might favour the infection of new hosts within the same tick population, thus increasing their mortality rate. Interestingly, *B. bassiana* negatively affected the reproductive efficiency and fitness indexes of the engorged infected females thus suggesting that the fungus may propagate within ticks at different developmental stages. In addition, *B. bassiana* reduced the larval and nymphal moulting rates, inhibited the hatching of eggs directly infected, eventually increasing its efficacy as an anti-tick agent.

## Conclusions

This study demonstrates that native strains of *B. bassiana* are highly virulent towards all life-cycle developmental stages of *R. sanguineus* s.l., thus suggesting that the use of this fungus may be effective in controlling tick populations in the environment. Nonetheless, further laboratory and field studies are required to determine the best route to the application and frequency of treatment for using this fungus as a bio-control agent. In addition, this fungus could also be used in combination with chemicals for the control of tick infestations in animal shelters, toward reducing the hazards related to the excessive use of chemical products.
